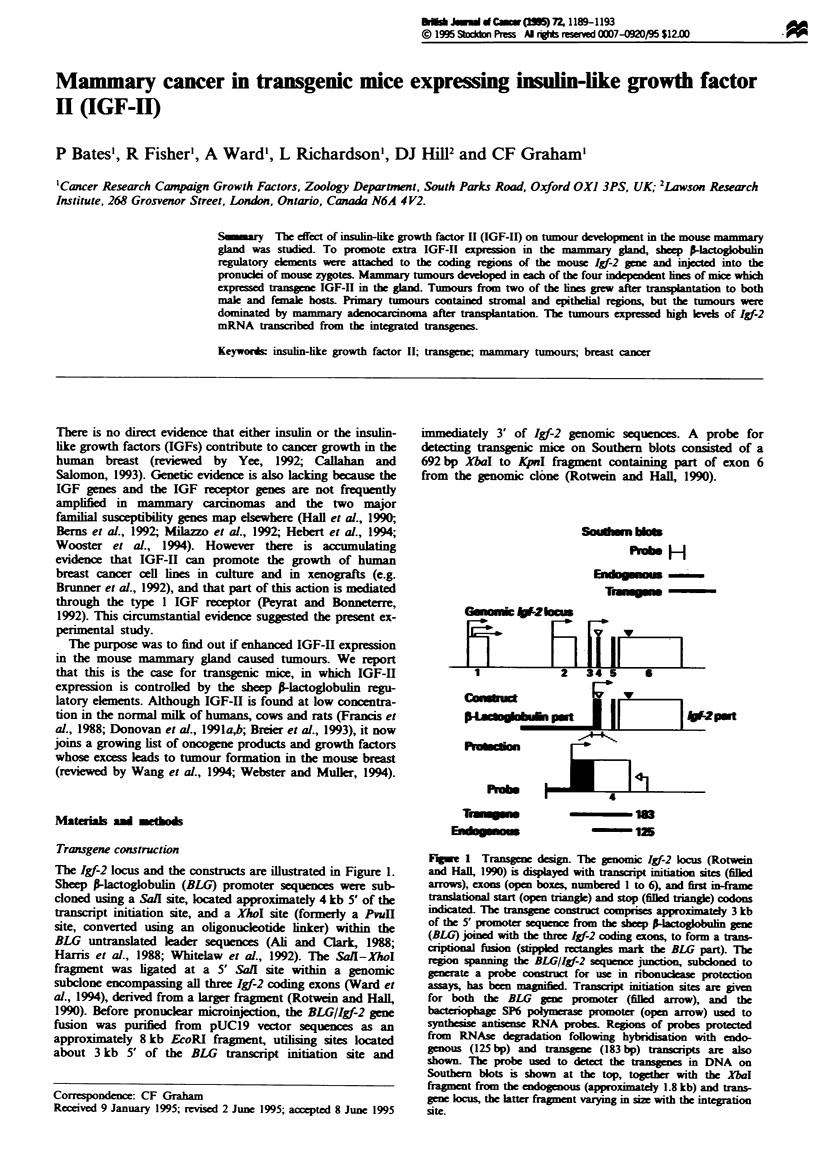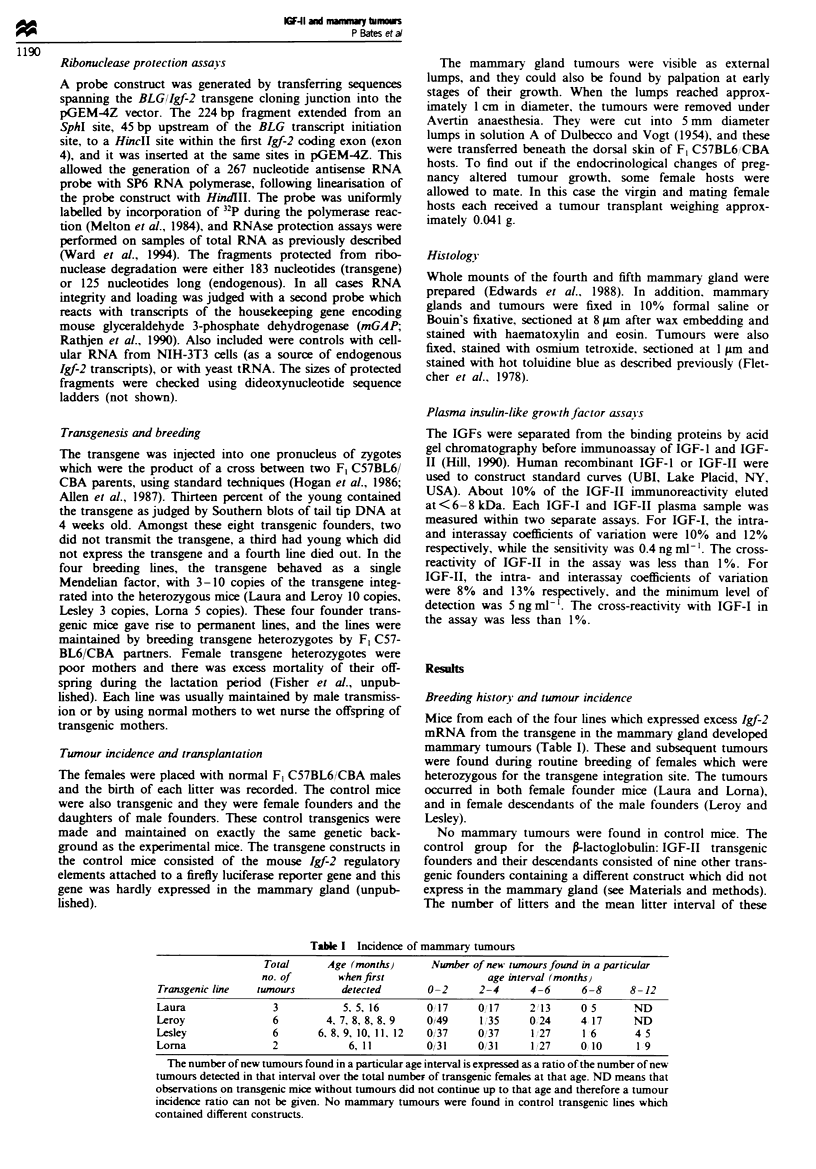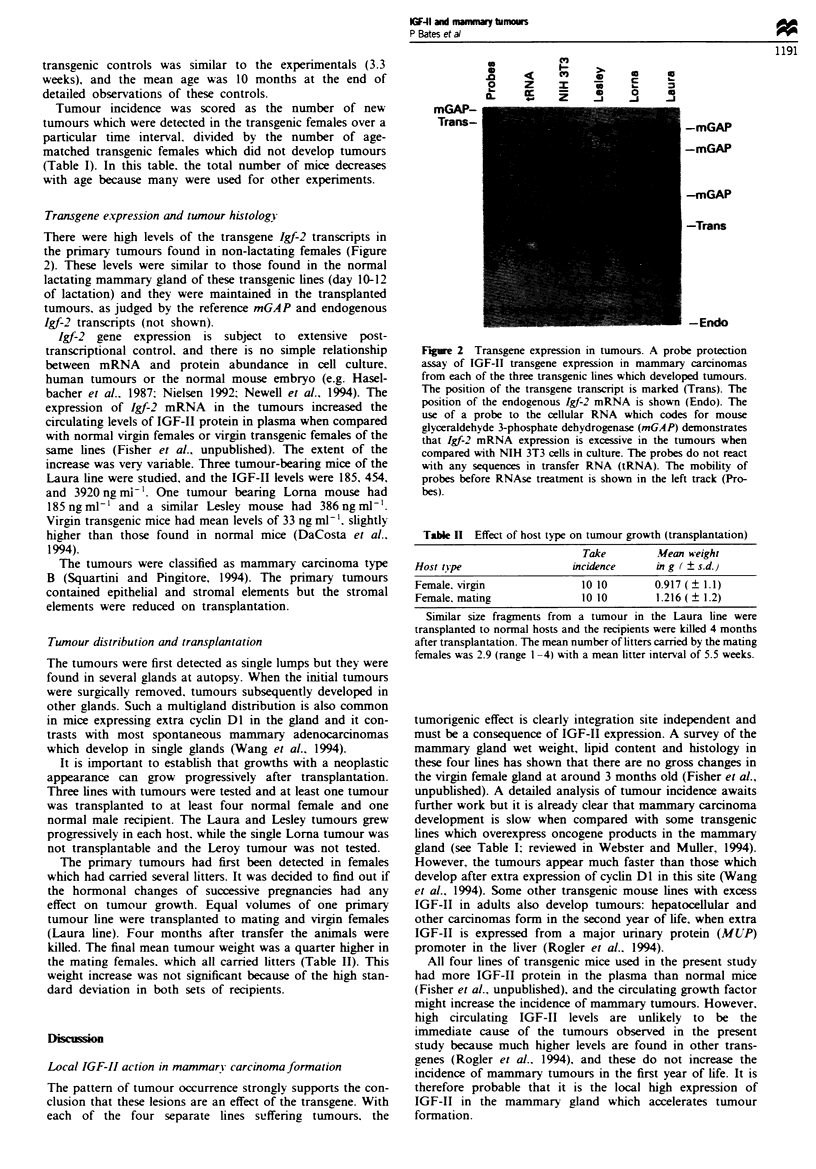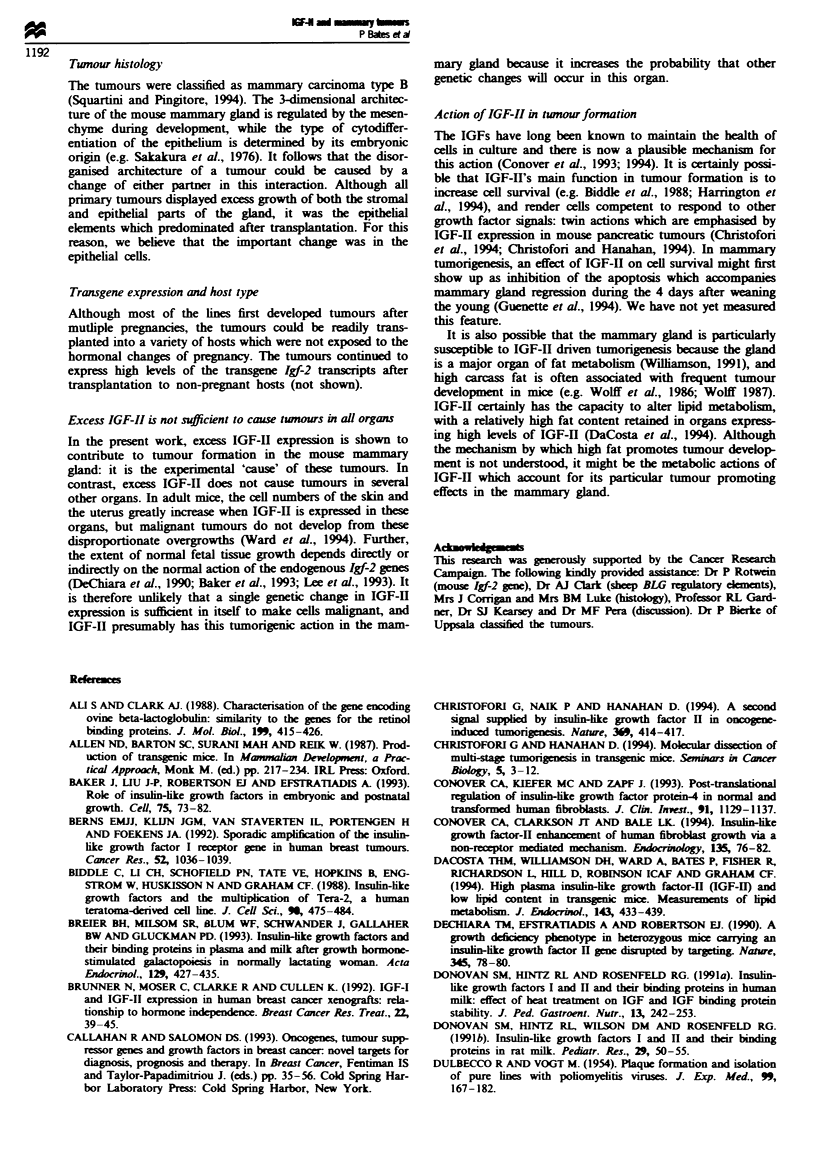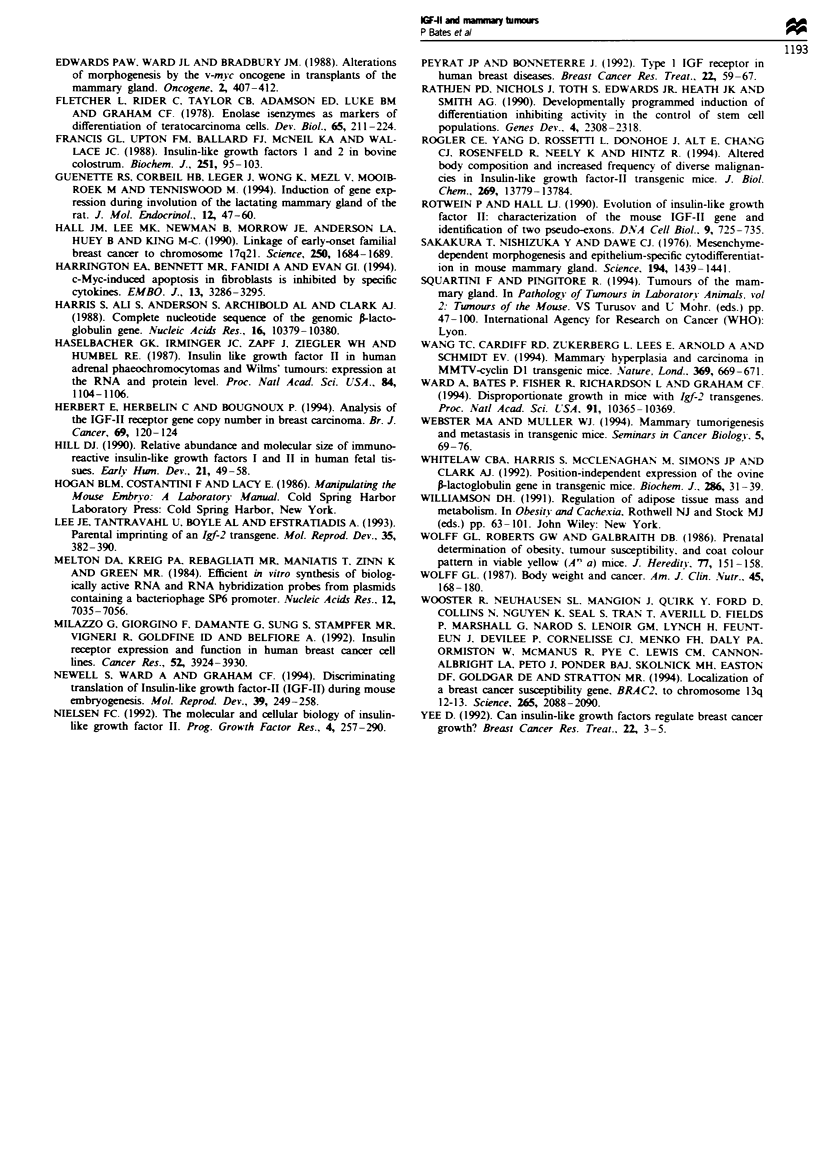# Mammary cancer in transgenic mice expressing insulin-like growth factor II (IGF-II)

**DOI:** 10.1038/bjc.1995.484

**Published:** 1995-11

**Authors:** P. Bates, R. Fisher, A. Ward, L. Richardson, D. J. Hill, C. F. Graham

**Affiliations:** Cancer Research Campaign Growth Factors, Zoology Department, Oxford, UK.

## Abstract

**Images:**


© 1995 Stockton Press All rights reserved 0007–0920/95 $12.00

### Mammary cancer in transgenic mice expressing insulin-like growth factor II (IGF-II)

P Bates[1], R Fisher[1], A Ward[1], L Richardson[1], DJ Hill[2] and CF Graham[1]

[1]*Cancer Research Campaign Growth Factors, Zoology Department, South Parks Road, Oxford OX1 3PS, UK;* [2]*Lawson Research Institute, 268 Grosvenor Street, London, Ontario, Canada N6A 4V2.*

**Summary** The effect of insulin-like growth factor II (IGF-II) on tumour development in the mouse mammary gland was studied. To promote extra IGF-II expression in the mammary gland, sheep β-lactoglobulin regulatory elements were attached to the coding regions of the mouse *Igf-2* gene and injected into the pronuclei of mouse zygotes. Mammary tumours developed in each of the four independent lines of mice which expressed transgene IGF-II in the gland. Tumours from two of the lines grew after transplantation to both male and female hosts. Primary tumours contained stromal and epithelial regions, but the tumours were dominated by mammary adenocarcinoma after transplantation. The tumours expressed high levels of *Igf-2* mRNA transcribed from the integrated transgenes.

**Keywords:** insulin-like growth factor II; transgene; mammary tumours; breast cancer

There is no direct evidence that either insulin or the insulin-like growth factors (IGFs) contribute to cancer growth in the human breast (reviewed by Yee, 1992; Callahan and Salomon, 1993). Genetic evidence is also lacking because the IGF genes and the IGF receptor genes are not frequently amplified in mammary carcinomas and the two major familial susceptibility genes map elsewhere (Hall *et al*., 1990; Berns *et al*., 1992; Milazzo *et al*., 1992; Hebert *et al*., 1994; Wooster *et al*., 1994). However there is accumulating evidence that IGF-II can promote the growth of human breast cancer cell lines in culture and in xenografts (e.g. Brunner *et al*., 1992), and that part of this action is mediated through the type 1 IGF receptor (Peyrat and Bonneterre, 1992). This circumstantial evidence suggested the present experimental study.

The purpose was to find out if enhanced IGF-II expression in the mouse mammary gland caused tumours. We report that this is the case for transgenic mice, in which IGF-II expression is controlled by the sheep β-lactoglobulin regulatory elements. Although IGF-II is found at low concentration in the normal milk of humans, cows and rats (Francis *et al*., 1988; Donovan *et al*., 1991*a*,*b*; Breier *et al*., 1993), it now joins a growing list of oncogene products and growth factors whose excess leads to tumour formation in the mouse breast (reviewed by Wang *et al*., 1994; Webster and Muller, 1994).

### Materials and methods

### *Transgene construction*

The *Igf-2* locus and the constructs are illustrated in Figure 1. Sheep β-lactoglobulin (*BLG*) promoter sequences were subcloned using a *Sal*I site, located approximately 4 kb 5′ of the transcript initiation site, and a *Xho*I site (formerly a *Pvu*II site, converted using an oligonucleotide linker) within the *BLG* untranslated leader sequences (Ali and Clark, 1988; Harris *et al*., 1988; Whitelaw *et al*., 1992). The *Sal*I–*Xho*I fragment was ligated at a 5′ *Sal*I site within a genomic subclone encompassing all three *Igf-2* coding exons (Ward *et al*., 1994), derived from a larger fragment (Rotwein and Hall, 1990). Before pronuclear microinjection, the *BLG*/*Igf-2* gene fusion was purified from pUC19 vector sequences as an approximately 8 kb *Eco*RI fragment, utilising sites located about 3 kb 5′ of the *BLG* transcript initiation site and immediately 3′ of *Igf-2* genomic sequences. A probe for detecting transgenic mice on Southern blots consisted of a 692 bp *Xba*I to *Kpn*I fragment containing part of exon 6 from the genomic clone (Rotwein and Hall, 1990).

**Figure 1** Transgene design. The genomic *Igf-2* locus (Rotwein and Hall, 1990) is displayed with transcript initiation sites (filled arrows), exons (open boxes, numbered 1 to 6), and first in-frame translational start (open triangle) and stop (filled triangle) codons indicated. The transgene construct comprises approximately 3 kb of the 5′ promoter sequence from the sheep β-lactoglobulin gene (*BLG*) joined with the three *Igf-2* coding exons, to form a transcriptional fusion (stippled rectangles mark the *BLG* part). The region spanning the *BLG*/*Igf-2* sequence junction, subcloned to generate a probe construct for use in ribonuclease protection assays, has been magnified. Transcript initiation sites are given for both the *BLG* gene promoter (filled arrow), and the bacteriophage SP6 polymerase promoter (open arrow) used to synthesise antisense RNA probes. Regions of probes protected from RNAse degradation following hybridisation with endogenous (125 bp) and transgene (183 bp) transcripts are also shown. The probe used to detect the transgenes in DNA on Southern blots is shown at the top, together with the *Xba*I fragment from the endogenous (approximately 1.8 kb) and transgene locus, the latter fragment varying in size with the integration site.

Correspondence: CF Graham
Received 9 January 1995; revised 2 June 1995; accepted 8 June 1995

*Ribonuclease protection assays*

A probe construct was generated by transferring sequences spanning the *BLG/Igf-2* transgene cloning junction into the pGEM-4Z vector. The 224 bp fragment extended from an *Sph*I site, 45 bp upstream of the *BLG* transcript initiation site, to a *Hinc*II site within the first *Igf-2* coding exon (exon 4), and it was inserted at the same sites in pGEM-4Z. This allowed the generation of a 267 nucleotide antisense RNA probe with SP6 RNA polymerase, following linearisation of the probe construct with *Hind*III. The probe was uniformly labelled by incorporation of $^{32}P$ during the polymerase reaction (Melton *et al.*, 1984), and RNAse protection assays were performed on samples of total RNA as previously described (Ward *et al.*, 1994). The fragments protected from ribonuclease degradation were either 183 nucleotides (transgene) or 125 nucleotides long (endogenous). In all cases RNA integrity and loading was judged with a second probe which reacts with transcripts of the housekeeping gene encoding mouse glyceraldehyde 3-phosphate dehydrogenase (*mGAP*; Rathjen *et al.*, 1990). Also included were controls with cellular RNA from NIH-3T3 cells (as a source of endogenous *Igf-2* transcripts), or with yeast tRNA. The sizes of protected fragments were checked using dideoxynucleotide sequence ladders (not shown).

*Transgenesis and breeding*

The transgene was injected into one pronucleus of zygotes which were the product of a cross between two $F_1$ C57BL6/CBA parents, using standard techniques (Hogan *et al.*, 1986; Allen *et al.*, 1987). Thirteen percent of the young contained the transgene as judged by Southern blots of tail tip DNA at 4 weeks old. Amongst these eight transgenic founders, two did not transmit the transgene, a third had young which did not express the transgene and a fourth line died out. In the four breeding lines, the transgene behaved as a single Mendelian factor, with 3–10 copies of the transgene integrated into the heterozygous mice (Laura and Leroy 10 copies, Lesley 3 copies, Lorna 5 copies). These four founder transgenic mice gave rise to permanent lines, and the lines were maintained by breeding transgene heterozygotes by $F_1$ C57-BL6/CBA partners. Female transgene heterozygotes were poor mothers and there was excess mortality of their offspring during the lactation period (Fisher *et al.*, unpublished). Each line was usually maintained by male transmission or by using normal mothers to wet nurse the offspring of transgenic mothers.

*Tumour incidence and transplantation*

The females were placed with normal $F_1$ C57BL6/CBA males and the birth of each litter was recorded. The control mice were also transgenic and they were female founders and the daughters of male founders. These control transgenics were made and maintained on exactly the same genetic background as the experimental mice. The transgene constructs in the control mice consisted of the mouse *Igf-2* regulatory elements attached to a firefly luciferase reporter gene and this gene was hardly expressed in the mammary gland (unpublished).

The mammary gland tumours were visible as external lumps, and they could also be found by palpation at early stages of their growth. When the lumps reached approximately 1 cm in diameter, the tumours were removed under Avertin anaesthesia. They were cut into 5 mm diameter lumps in solution A of Dulbecco and Vogt (1954), and these were transferred beneath the dorsal skin of $F_1$ C57BL6/CBA hosts. To find out if the endocrinological changes of pregnancy altered tumour growth, some female hosts were allowed to mate. In this case the virgin and mating female hosts each received a tumour transplant weighing approximately 0.041 g.

*Histology*

Whole mounts of the fourth and fifth mammary gland were prepared (Edwards *et al.*, 1988). In addition, mammary glands and tumours were fixed in 10% formal saline or Bouin's fixative, sectioned at 8 μm after wax embedding and stained with haematoxylin and eosin. Tumours were also fixed, stained with osmium tetroxide, sectioned at 1 μm and stained with hot toluidine blue as described previously (Fletcher *et al.*, 1978).

*Plasma insulin-like growth factor assays*

The IGFs were separated from the binding proteins by acid gel chromatography before immunoassay of IGF-1 and IGF-II (Hill, 1990). Human recombinant IGF-1 or IGF-II were used to construct standard curves (UBI, Lake Placid, NY, USA). About 10% of the IGF-II immunoreactivity eluted at $<$6–8 kDa. Each IGF-I and IGF-II plasma sample was measured within two separate assays. For IGF-I, the intra- and interassay coefficients of variation were 10% and 12% respectively, while the sensitivity was 0.4 ng ml$^{-1}$. The cross-reactivity of IGF-II in the assay was less than 1%. For IGF-II, the intra- and interassay coefficients of variation were 8% and 13% respectively, and the minimum level of detection was 5 ng ml$^{-1}$. The cross-reactivity with IGF-I in the assay was less than 1%.

### Results

*Breeding history and tumour incidence*

Mice from each of the four lines which expressed excess *Igf-2* mRNA from the transgene in the mammary gland developed mammary tumours (Table I). These and subsequent tumours were found during routine breeding of females which were heterozygous for the transgene integration site. The tumours occurred in both female founder mice (Laura and Lorna), and in female descendants of the male founders (Leroy and Lesley).

No mammary tumours were found in control mice. The control group for the β-lactoglobulin: IGF-II transgenic founders and their descendants consisted of nine other transgenic founders containing a different construct which did not express in the mammary gland (see Materials and methods). The number of litters and the mean litter interval of these

**Table I** Incidence of mammary tumours

| *Transgenic line* | *Total no. of tumours* | *Age (months) when first detected* | *Number of new tumours found in a particular age interval (months)* 0–2 | 2–4 | 4–6 | 6–8 | 8–12 |
|---|---|---|---|---|---|---|---|
| Laura | 3 | 5, 5, 16 | 0/17 | 0/17 | 2/13 | 0/5 | ND |
| Leroy | 6 | 4, 7, 8, 8, 8, 9 | 0/49 | 1/35 | 0/24 | 4/17 | ND |
| Lesley | 6 | 6, 8, 9, 10, 11, 12 | 0/37 | 0/37 | 1/27 | 1/6 | 4/5 |
| Lorna | 2 | 6, 11 | 0/31 | 0/31 | 1/27 | 0/10 | 1/9 |

The number of new tumours found in a particular age interval is expressed as a ratio of the number of new tumours detected in that interval over the total number of transgenic females at that age. ND means that observations on transgenic mice without tumours did not continue up to that age and therefore a tumour incidence ratio can not be given. No mammary tumours were found in control transgenic lines which contained different constructs.

transgenic controls was similar to the experimentals (3.3 weeks), and the mean age was 10 months at the end of detailed observations of these controls.

Tumour incidence was scored as the number of new tumours which were detected in the transgenic females over a particular time interval, divided by the number of age-matched transgenic females which did not develop tumours (Table I). In this table, the total number of mice decreases with age because many were used for other experiments.

### *Transgene expression and tumour histology*

There were high levels of the transgene *Igf-2* transcripts in the primary tumours found in non-lactating females (Figure 2). These levels were similar to those found in the normal lactating mammary gland of these transgenic lines (day 10-12 of lactation) and they were maintained in the transplanted tumours, as judged by the reference *mGAP* and endogenous *Igf-2* transcripts (not shown).

*Igf-2* gene expression is subject to extensive post-transcriptional control, and there is no simple relationship between mRNA and protein abundance in cell culture, human tumours or the normal mouse embryo (e.g. Haselbacher *et al*., 1987; Nielsen 1992; Newell *et al*., 1994). The expression of *Igf-2* mRNA in the tumours increased the circulating levels of IGF-II protein in plasma when compared with normal virgin females or virgin transgenic females of the same lines (Fisher *et al*., unpublished). The extent of the increase was very variable. Three tumour-bearing mice of the Laura line were studied, and the IGF-II levels were 185, 454, and 3920 ng $ml^{-1}$. One tumour bearing Lorna mouse had 185 ng $ml^{-1}$ and a similar Lesley mouse had 386 ng $ml^{-1}$. Virgin transgenic mice had mean levels of 33 ng $ml^{-1}$, slightly higher than those found in normal mice (DaCosta *et al*., 1994).

The tumours were classified as mammary carcinoma type B (Squartini and Pingitore, 1994). The primary tumours contained epithelial and stromal elements but the stromal elements were reduced on transplantation.

### *Tumour distribution and transplantation*

The tumours were first detected as single lumps but they were found in several glands at autopsy. When the initial tumours were surgically removed, tumours subsequently developed in other glands. Such a multigland distribution is also common in mice expressing extra cyclin D1 in the gland and it contrasts with most spontaneous mammary adenocarcinomas which develop in single glands (Wang *et al*., 1994).

It is important to establish that growths with a neoplastic appearance can grow progressively after transplantation. Three lines with tumours were tested and at least one tumour was transplanted to at least four normal female and one normal male recipient. The Laura and Lesley tumours grew progressively in each host, while the single Lorna tumour was not transplantable and the Leroy tumour was not tested.

The primary tumours had first been detected in females which had carried several litters. It was decided to find out if the hormonal changes of successive pregnancies had any effect on tumour growth. Equal volumes of one primary tumour line were transplanted to mating and virgin females (Laura line). Four months after transfer the animals were killed. The final mean tumour weight was a quarter higher in the mating females, which all carried litters (Table II). This weight increase was not significant because of the high standard deviation in both sets of recipients.

**Figure 2** Transgene expression in tumours. A probe protection assay of IGF-II transgene expression in mammary carcinomas from each of the three transgenic lines which developed tumours. The position of the transgene transcript is marked (Trans). The position of the endogenous *Igf-2* mRNA is shown (Endo). The use of a probe to the cellular RNA which codes for mouse glyceraldehyde 3-phosphate dehydrogenase (*mGAP*) demonstrates that *Igf-2* mRNA expression is excessive in the tumours when compared with NIH 3T3 cells in culture. The probes do not react with any sequences in transfer RNA (tRNA). The mobility of probes before RNAse treatment is shown in the left track (Probes).

**Table II** Effect of host type on tumour growth (transplantation)

| *Host type* | *Take incidence* | *Mean weight in g ( ± s.d.)* |
|---|---|---|
| Female, virgin | 10 10 | 0.917 ( ± 1.1) |
| Female, mating | 10 10 | 1.216 ( ± 1.2) |

Similar size fragments from a tumour in the Laura line were transplanted to normal hosts and the recipients were killed 4 months after transplantation. The mean number of litters carried by the mating females was 2.9 (range 1–4) with a mean litter interval of 5.5 weeks.

### Discussion

### *Local IGF-II action in mammary carcinoma formation*

The pattern of tumour occurrence strongly supports the conclusion that these lesions are an effect of the transgene. With each of the four separate lines suffering tumours, the tumorigenic effect is clearly integration site independent and must be a consequence of IGF-II expression. A survey of the mammary gland wet weight, lipid content and histology in these four lines has shown that there are no gross changes in the virgin female gland at around 3 months old (Fisher *et al*., unpublished). A detailed analysis of tumour incidence awaits further work but it is already clear that mammary carcinoma development is slow when compared with some transgenic lines which overexpress oncogene products in the mammary gland (see Table I; reviewed in Webster and Muller, 1994). However, the tumours appear much faster than those which develop after extra expression of cyclin D1 in this site (Wang *et al*., 1994). Some other transgenic mouse lines with excess IGF-II in adults also develop tumours: hepatocellular and other carcinomas form in the second year of life, when extra IGF-II is expressed from a major urinary protein (*MUP*) promoter in the liver (Rogler *et al*., 1994).

All four lines of transgenic mice used in the present study had more IGF-II protein in the plasma than normal mice (Fisher *et al*., unpublished), and the circulating growth factor might increase the incidence of mammary tumours. However, high circulating IGF-II levels are unlikely to be the immediate cause of the tumours observed in the present study because much higher levels are found in other transgenes (Rogler *et al*., 1994), and these do not increase the incidence of mammary tumours in the first year of life. It is therefore probable that it is the local high expression of IGF-II in the mammary gland which accelerates tumour formation.

*Tumour histology*

The tumours were classified as mammary carcinoma type B (Squartini and Pingitore, 1994). The 3-dimensional architecture of the mouse mammary gland is regulated by the mesenchyme during development, while the type of cytodifferentiation of the epithelium is determined by its embryonic origin (e.g. Sakakura *et al.*, 1976). It follows that the disorganised architecture of a tumour could be caused by a change of either partner in this interaction. Although all primary tumours displayed excess growth of both the stromal and epithelial parts of the gland, it was the epithelial elements which predominated after transplantation. For this reason, we believe that the important change was in the epithelial cells.

*Transgene expression and host type*

Although most of the lines first developed tumours after mutliple pregnancies, the tumours could be readily transplanted into a variety of hosts which were not exposed to the hormonal changes of pregnancy. The tumours continued to express high levels of the transgene *Igf-2* transcripts after transplantation to non-pregnant hosts (not shown).

*Excess IGF-II is not sufficient to cause tumours in all organs*

In the present work, excess IGF-II expression is shown to contribute to tumour formation in the mouse mammary gland: it is the experimental 'cause' of these tumours. In contrast, excess IGF-II does not cause tumours in several other organs. In adult mice, the cell numbers of the skin and the uterus greatly increase when IGF-II is expressed in these organs, but malignant tumours do not develop from these disproportionate overgrowths (Ward *et al.*, 1994). Further, the extent of normal fetal tissue growth depends directly or indirectly on the normal action of the endogenous *Igf-2* genes (DeChiara *et al.*, 1990; Baker *et al.*, 1993; Lee *et al.*, 1993). It is therefore unlikely that a single genetic change in IGF-II expression is sufficient in itself to make cells malignant, and IGF-II presumably has this tumorigenic action in the mammary gland because it increases the probability that other genetic changes will occur in this organ.

*Action of IGF-II in tumour formation*

The IGFs have long been known to maintain the health of cells in culture and there is now a plausible mechanism for this action (Conover *et al.*, 1993; 1994). It is certainly possible that IGF-II's main function in tumour formation is to increase cell survival (e.g. Biddle *et al.*, 1988; Harrington *et al.*, 1994), and render cells competent to respond to other growth factor signals: twin actions which are emphasised by IGF-II expression in mouse pancreatic tumours (Christofori *et al.*, 1994; Christofori and Hanahan, 1994). In mammary tumorigenesis, an effect of IGF-II on cell survival might first show up as inhibition of the apoptosis which accompanies mammary gland regression during the 4 days after weaning the young (Guenette *et al.*, 1994). We have not yet measured this feature.

It is also possible that the mammary gland is particularly susceptible to IGF-II driven tumorigenesis because the gland is a major organ of fat metabolism (Williamson, 1991), and high carcass fat is often associated with frequent tumour development in mice (e.g. Wolff *et al.*, 1986; Wolff 1987). IGF-II certainly has the capacity to alter lipid metabolism, with a relatively high fat content retained in organs expressing high levels of IGF-II (DaCosta *et al.*, 1994). Although the mechanism by which high fat promotes tumour development is not understood, it might be the metabolic actions of IGF-II which account for its particular tumour promoting effects in the mammary gland.

**Acknowledgements**

This research was generously supported by the Cancer Research Campaign. The following kindly provided assistance: Dr P Rotwein (mouse *Igf-2* gene), Dr AJ Clark (sheep *BLG* regulatory elements), Mrs J Corrigan and Mrs BM Luke (histology), Professor RL Gardner, Dr SJ Kearsey and Dr MF Pera (discussion). Dr P Bierke of Uppsala classified the tumours.

**References**

ALI S AND CLARK AJ. (1988). Characterisation of the gene encoding ovine beta-lactoglobulin: similarity to the genes for the retinol binding proteins. *J. Mol. Biol.*, **199**, 415-426.

ALLEN ND, BARTON SC, SURANI MAH AND REIK W. (1987). Production of transgenic mice. In *Mammalian Development, a Practical Approach*, Monk M. (ed.) pp. 217-234. IRL Press: Oxford.

BAKER J, LIU J-P, ROBERTSON EJ AND EFSTRATIADIS A. (1993). Role of insulin-like growth factors in embryonic and postnatal growth. *Cell*, **75**, 73-82.

BERNS EMJJ, KLIJN JGM, VAN STAVERTEN IL, PORTENGEN H AND FOEKENS JA. (1992). Sporadic amplification of the insulin-like growth factor I receptor gene in human breast tumours. *Cancer Res.*, **52**, 1036-1039.

BIDDLE C, LI CH, SCHOFIELD PN, TATE VE, HOPKINS B, ENGSTROM W, HUSKISSON N AND GRAHAM CF. (1988). Insulin-like growth factors and the multiplication of Tera-2, a human teratoma-derived cell line. *J. Cell Sci.*, **90**, 475-484.

BREIER BH, MILSOM SR, BLUM WF, SCHWANDER J, GALLAHER BW AND GLUCKMAN PD. (1993). Insulin-like growth factors and their binding proteins in plasma and milk after growth hormone-stimulated galactopoiesis in normally lactating woman. *Acta Endocrinol.*, **129**, 427-435.

BRUNNER N, MOSER C, CLARKE R AND CULLEN K. (1992). IGF-I and IGF-II expression in human breast cancer xenografts: relationship to hormone independence. *Breast Cancer Res. Treat.*, **22**, 39-45.

CALLAHAN R AND SALOMON DS. (1993). Oncogenes, tumour suppressor genes and growth factors in breast cancer: novel targets for diagnosis, prognosis and therapy. In *Breast Cancer*, Fentiman IS and Taylor-Papadimitriou J. (eds.) pp. 35-56. Cold Spring Harbor Laboratory Press: Cold Spring Harbor, New York.

CHRISTOFORI G, NAIK P AND HANAHAN D. (1994). A second signal supplied by insulin-like growth factor II in oncogene-induced tumorigenesis. *Nature*, **369**, 414-417.

CHRISTOFORI G AND HANAHAN D. (1994). Molecular dissection of multi-stage tumorigenesis in transgenic mice. *Seminars in Cancer Biology*, **5**, 3-12.

CONOVER CA, KIEFER MC AND ZAPF J. (1993). Post-translational regulation of insulin-like growth factor protein-4 in normal and transformed human fibroblasts. *J. Clin. Invest.*, **91**, 1129-1137.

CONOVER CA, CLARKSON JT AND BALE LK. (1994). Insulin-like growth factor-II enhancement of human fibroblast growth via a non-receptor mediated mechanism. *Endocrinology*, **135**, 76-82.

DACOSTA THM, WILLIAMSON DH, WARD A, BATES P, FISHER R, RICHARDSON L, HILL D, ROBINSON ICAF AND GRAHAM CF. (1994). High plasma insulin-like growth factor-II (IGF-II) and low lipid content in transgenic mice. Measurements of lipid metabolism. *J. Endocrinol.*, **143**, 433-439.

DECHIARA TM, EFSTRATIADIS A AND ROBERTSON EJ. (1990). A growth deficiency phenotype in heterozygous mice carrying an insulin-like growth factor II gene disrupted by targeting. *Nature*, **345**, 78-80.

DONOVAN SM, HINTZ RL AND ROSENFELD RG. (1991*a*). Insulin-like growth factors I and II and their binding proteins in human milk: effect of heat treatment on IGF and IGF binding protein stability. *J. Ped. Gastroent. Nutr.*, **13**, 242-253.

DONOVAN SM, HINTZ RL, WILSON DM AND ROSENFELD RG. (1991*b*). Insulin-like growth factors I and II and their binding proteins in rat milk. *Pediatr. Res.*, **29**, 50-55.

DULBECCO R AND VOGT M. (1954). Plaque formation and isolation of pure lines with poliomyelitis viruses. *J. Exp. Med.*, **99**, 167-182.

EDWARDS PAW, WARD JL AND BRADBURY JM. (1988). Alterations of morphogenesis by the v-*myc* oncogene in transplants of the mammary gland. *Oncogene*, **2**, 407-412.

FLETCHER L, RIDER C, TAYLOR CB, ADAMSON ED, LUKE BM AND GRAHAM CF. (1978). Enolase isenzymes as markers of differentiation of teratocarcinoma cells. *Dev. Biol.*, **65**, 211-224.

FRANCIS GL, UPTON FM, BALLARD FJ, MCNEIL KA AND WALLACE JC. (1988). Insulin-like growth factors 1 and 2 in bovine colostrum. *Biochem. J.*, **251**, 95-103.

GUENETTE RS, CORBEIL HB, LEGER J, WONG K, MEZL V, MOOIBROEK M AND TENNISWOOD M. (1994). Induction of gene expression during involution of the lactating mammary gland of the rat. *J. Mol. Endocrinol.*, **12**, 47-60.

HALL JM, LEE MK, NEWMAN B, MORROW JE, ANDERSON LA, HUEY B AND KING M-C. (1990). Linkage of early-onset familial breast cancer to chromosome 17q21. *Science*, **250**, 1684-1689.

HARRINGTON EA, BENNETT MR, FANIDI A AND EVAN GI. (1994). c-Myc-induced apoptosis in fibroblasts is inhibited by specific cytokines. *EMBO. J.*, **13**, 3286-3295.

HARRIS S, ALI S, ANDERSON S, ARCHIBOLD AL AND CLARK AJ. (1988). Complete nucleotide sequence of the genomic β-lactoglobulin gene. *Nucleic Acids Res.*, **16**, 10379-10380.

HASELBACHER GK, IRMINGER JC, ZAPF J, ZIEGLER WH AND HUMBEL RE. (1987). Insulin like growth factor II in human adrenal phaeochromocytomas and Wilms' tumours: expression at the RNA and protein level. *Proc. Natl Acad. Sci. USA.*, **84**, 1104-1106.

HERBERT E, HERBELIN C AND BOUGNOUX P. (1994). Analysis of the IGF-II receptor gene copy number in breast carcinoma. *Br. J. Cancer*, **69**, 120-124

HILL DJ. (1990). Relative abundance and molecular size of immunoreactive insulin-like growth factors I and II in human fetal tissues. *Early Hum. Dev.*, **21**, 49-58.

HOGAN BLM, COSTANTINI F AND LACY E. (1986). *Manipulating the Mouse Embryo: A Laboratory Manual.* Cold Spring Harbor Laboratory Press: Cold Spring Harbor, New York.

LEE JE, TANTRAVAHL U, BOYLE AL AND EFSTRATIADIS A. (1993). Parental imprinting of an *Igf-2* transgene. *Mol. Reprod. Dev.*, **35**, 382-390.

MELTON DA, KREIG PA, REBAGLIATI MR, MANIATIS T, ZINN K AND GREEN MR. (1984). Efficient *in vitro* synthesis of biologically active RNA and RNA hybridization probes from plasmids containing a bacteriophage SP6 promoter. *Nucleic Acids Res.*, **12**, 7035-7056.

MILAZZO G, GIORGINO F, DAMANTE G, SUNG S, STAMPFER MR, VIGNERI R, GOLDFINE ID AND BELFIORE A. (1992). Insulin receptor expression and function in human breast cancer cell lines. *Cancer Res.*, **52**, 3924-3930.

NEWELL S, WARD A AND GRAHAM CF. (1994). Discriminating translation of Insulin-like growth factor-II (IGF-II) during mouse embryogenesis. *Mol. Reprod. Dev.*, **39**, 249-258.

NIELSEN FC. (1992). The molecular and cellular biology of insulin-like growth factor II. *Prog. Growth Factor Res.*, **4**, 257-290.

PEYRAT JP AND BONNETERRE J. (1992). Type 1 IGF receptor in human breast diseases. *Breast Cancer Res. Treat.*, **22**, 59-67.

RATHJEN PD, NICHOLS J, TOTH S, EDWARDS JR, HEATH JK AND SMITH AG. (1990). Developmentally programmed induction of differentiation inhibiting activity in the control of stem cell populations. *Genes Dev.*, **4**, 2308-2318.

ROGLER CE, YANG D, ROSSETTI L, DONOHOE J, ALT E, CHANG CJ, ROSENFELD R, NEELY K AND HINTZ R. (1994). Altered body composition and increased frequency of diverse malignancies in Insulin-like growth factor-II transgenic mice. *J. Biol. Chem.*, **269**, 13779-13784.

ROTWEIN P AND HALL LJ. (1990). Evolution of insulin-like growth factor II: characterization of the mouse IGF-II gene and identification of two pseudo-exons. *DNA Cell Biol.*, **9**, 725-735.

SAKAKURA T, NISHIZUKA Y AND DAWE CJ. (1976). Mesenchyme-dependent morphogenesis and epithelium-specific cytodifferentiation in mouse mammary gland. *Science*, **194**, 1439-1441.

SQUARTINI F AND PINGITORE R. (1994). Tumours of the mammary gland. In *Pathology of Tumours in Laboratory Animals, vol 2: Tumours of the Mouse*. VS Turusov and U Mohr. (eds.) pp. 47-100. International Agency for Research on Cancer (WHO): Lyon.

WANG TC, CARDIFF RD, ZUKERBERG L, LEES E, ARNOLD A AND SCHMIDT EV. (1994). Mammary hyperplasia and carcinoma in MMTV-cyclin D1 transgenic mice. *Nature, Lond.*, **369**, 669-671.

WARD A, BATES P, FISHER R, RICHARDSON L AND GRAHAM CF. (1994). Disproportionate growth in mice with *Igf-2* transgenes. *Proc. Natl Acad. Sci. USA*, **91**, 10365-10369.

WEBSTER MA AND MULLER WJ. (1994). Mammary tumorigenesis and metastasis in transgenic mice. *Seminars in Cancer Biology*, **5**, 69-76.

WHITELAW CBA, HARRIS S, MCCLENAGHAN M, SIMONS JP AND CLARK AJ. (1992). Position-independent expression of the ovine β-lactoglobulin gene in transgenic mice. *Biochem. J.*, **286**, 31-39.

WILLIAMSON DH. (1991). Regulation of adipose tissue mass and metabolism. In *Obesity and Cachexia*. Rothwell NJ and Stock MJ (eds.) pp. 63-101. John Wiley: New York.

WOLFF GL, ROBERTS GW AND GALBRAITH DB. (1986). Prenatal determination of obesity, tumour susceptibility, and coat colour pattern in viable yellow ($A^{vy}$ *a*) mice. *J. Heredity*, **77**, 151-158.

WOLFF GL. (1987). Body weight and cancer. *Am. J. Clin. Nutr.*, **45**, 168-180.

WOOSTER R, NEUHAUSEN SL, MANGION J, QUIRK Y, FORD D, COLLINS N, NGUYEN K, SEAL S, TRAN T, AVERILL D, FIELDS P, MARSHALL G, NAROD S, LENOIR GM, LYNCH H, FEUNTEUN J, DEVILEE P, CORNELISSE CJ, MENKO FH, DALY PA, ORMISTON W, MCMANUS R, PYE C, LEWIS CM, CANNON-ALBRIGHT LA, PETO J, PONDER BAJ, SKOLNICK MH, EASTON DF, GOLDGAR DE AND STRATTON MR. (1994). Localization of a breast cancer susceptibility gene, *BRAC2*, to chromosome 13q 12-13. *Science*, **265**, 2088-2090.

YEE D. (1992). Can insulin-like growth factors regulate breast cancer growth? *Breast Cancer Res. Treat.*, **22**, 3-5.